# Cell-Free DNA Methylation Profiling Analysis—Technologies and Bioinformatics

**DOI:** 10.3390/cancers11111741

**Published:** 2019-11-06

**Authors:** Jinyong Huang, Liang Wang

**Affiliations:** Department of Tumor Biology, H. Lee Moffitt Cancer Center, Tampa, FL 33612, USA; Jinyong.huang@moffitt.org

**Keywords:** liquid biopsies, cell-free DNA, DNA methylation, bioinformatic, next-generation sequencing

## Abstract

Analysis of circulating nucleic acids in bodily fluids, referred to as “liquid biopsies”, is rapidly gaining prominence. Studies have shown that cell-free DNA (cfDNA) has great potential in characterizing tumor status and heterogeneity, as well as the response to therapy and tumor recurrence. DNA methylation is an epigenetic modification that plays an important role in a broad range of biological processes and diseases. It is well known that aberrant DNA methylation is generalizable across various samples and occurs early during the pathogenesis of cancer. Methylation patterns of cfDNA are also consistent with their originated cells or tissues. Systemic analysis of cfDNA methylation profiles has emerged as a promising approach for cancer detection and origin determination. In this review, we will summarize the technologies for DNA methylation analysis and discuss their feasibility for liquid biopsy applications. We will also provide a brief overview of the bioinformatic approaches for analysis of DNA methylation sequencing data. Overall, this review provides informative guidance for the selection of experimental and computational methods in cfDNA methylation-based studies.

## 1. Introduction

Cancer is one of the leading causes of death worldwide and the total number of diagnosed cancer cases keeps increasing globally [1]. Currently, tissue biopsies are the gold standards for cancer diagnosis as well as for molecular characterization. However, these conventional sampling methods have shown some limitations including difficulty in obtaining sufficient biomaterial, sampling bias arising from tumor genetic heterogeneity, and even procedural complications [2]. To address these issues, liquid biopsies, which are the analyses of circulating nucleic acids in blood or other body fluids, have emerged as a critical supplement to the tissue biopsies. Compared to tissue biopsies, liquid biopsies have several obvious advantages. First, the collection of blood is minimally invasive. Second, circulating nucleic acids in blood have a short half-life between 16 minutes and 2.5 hours, allowing liquid biopsies to be real-time and dynamic monitoring tools to estimate the tumor burden [3]. Third, since circulating nucleic acids can originate from different tissues, including metastatic tumor sites, liquid biopsies may represent a whole picture of a patient’s malignancy and solve the problem of tumor heterogeneity [4,5]. Recent studies have shown that liquid biopsies have a great potential to discover novel biomarkers for cancer diagnosis and prognosis [6,7,8]. Therefore, liquid biopsies have attracted great attention in recent years.

Cell-free DNA (cfDNA) in body fluids is a mixture of extracellular DNA fragments that are released from cells via apoptosis, necrosis, and active secretion [9]. The length of cfDNA is about 167 bp, corresponding to the unit size of a nucleosome [10,11]. Compared to healthy controls, a significantly higher level of cfDNA in cancer patients has been reported [12]. In addition, the increased cfDNA may decrease to the background level following surgery [13]. Results from these studies suggest that tumor-derived cfDNA is present in the blood of cancer patients. This tumor-derived cfDNA is called circulating tumor DNA (ctDNA). However, genetic analysis of ctDNA is very challenging because cfDNA is often limited in yield and highly fragmented [14]. Most importantly, ctDNA is extremely underrepresented in the high background of normal cfDNA [14]. The increasing availability and reliability of highly sensitive technologies, such as droplet digital PCR (ddPCR) and next-generation sequencing (NGS), are facilitating the development of liquid biopsies [15]. Currently, detections of cancer-related hotspot mutations have been approved for clinical applications. However, due to the limited number of recurrent mutations available for discriminating ctDNA from total cfDNA, application of these hot-spot detections is very limited [16,17]. On the other hand, aberrant DNA methylation occurs early during tumorigenesis and is abundantly present in the entire cancer process [18]. Therefore, it is believed that analysis of the ctDNA methylation pattern may be a more robust and sensitive approach for cancer diagnosis and prognosis [19].

**Figure 1:**
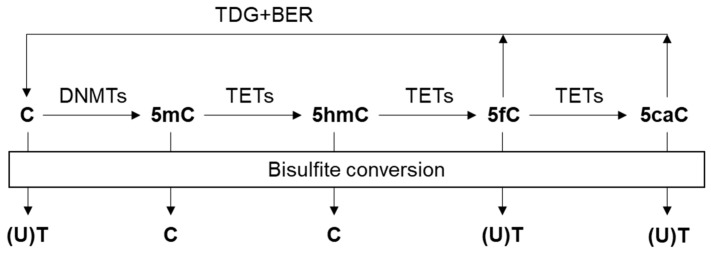
Cytosine variants and their products by bisulfite conversion. DNA methyltransferases (DNMTs) convert unmodified cytosine (C) to 5-methylcytosine (5mC) by adding a methyl group. Ten-eleven translocation (TET) enzymes oxidize 5mC to 5-hydroxymethylcytosine (5hmC), 5-formylcytosine (5fC) and 5-carboxylcytosine (5caC). Thymine DNA glycosylase (TDG) and the base excision repair (BER) pathway allow for regeneration of C from 5fC and 5caC. Upon bisulfite treatment, unmethylated cytosine (C) is deaminated to uracil (U) and eventually converted to thymine (T) via DNA amplification, while methylated C remains unaffected. 5hmC also protects C from deamination, while 5fC and 5caC do not.

DNA methylation is a common epigenetic modification achieved by adding a methyl group to the fifth carbon of cytosine (5-methylcytosine, 5mC) via DNA methyltransferases (DNMTs) (Figure 1). This modification occurs most frequently at cytosine residues in the sequence context of 5′-C-phosphate-G-3′ (CpG) [20]. The current human genome build contains about 28 million CpGs, 60–80% of which are methylated [21]. Generally, the majority of all CpGs are methylated in human, except short unmethylated regions called CpG islands (CGIs) [22]. On the contrary, the cancer genome is characterized by global hypomethylation and CGI-specific hypermethylation, resulting in genomic instability and transcriptional repression, respectively [23,24]. Growing evidence suggests that aberrant DNA methylation contributes to the tumorigenesis and tumor progression, which enables DNA methylation analysis as a promising approach for cancer detection [25,26,27,28]. Interestingly, the methylation patterns of cfDNA are consistent with the cells or tissues where they originate [29], implying that cfDNA methylation may serve as feasible and reliable cancer biomarkers [30,31]. 

## 2. Technologies for DNA Methylation Detection

### 2.1. Restriction Enzyme-Based Methods

The use of methylation restriction enzymes (MREs) to cleave DNA at a specific nucleotide sequence is a classical method for methylation study. Generally, two kinds of enzymes are used in this method. Methylation-sensitive enzymes cleave only unmethylated DNA and leave the methylated DNA intact, while methylation-insensitive enzymes can cleave regardless the methylation status of the recognition sites (see Reference [32] for all available MREs). Base on this principle, many array hybridization methods have been developed for detecting 5mC. For example, the HpaII-tiny fragment Enrichment by Ligation-mediated PCR (HELP) assay [33] and methyl-sensitive cut counting (MSCC) [34]. More recently, MRE digestion followed by sequencing (MRE-seq or methyl-seq) has been developed and used to study the role of DNA methylation in regulating alternative promoters [35,36]. After digestion, the fragmented DNA is directly sized-selected for library preparation without the need for sonication. However, due to the limited CpG-containing recognition sites, MRE-seq exhibits a lack of coverage of the whole methylome. Importantly, the severely fragmented nature of cfDNA restricts the application of MRE-seq for cfDNA methylation profiling as some restriction sites may have been destroyed. With the emergence of ddPCR, absolute quantification of DNA methylation based on MRE digestion has been developed [37]. Briefly, MRE is used to recognize methylated and unmethylated DNA. Then the methylation level is determined quantitatively on the microfluidic chip using Poisson statistics. Due to high sensitivity of ddPCR, this method allows ultra-low DNA input and therefore suitable for target-specific liquid biopsies. Moreover, it allows for a relatively easy primer and probe design as it is free from bisulfite conversion.

### 2.2. Bisulfite Conversion-Based Methods

Bisulfite conversion-based methods are regarded as the gold standard for DNA methylation study. Upon sodium bisulfite treatment on denatured DNA, unmethylated cytosine (C) residues are deaminated to uracil (U) and eventually converted to thymine (T) via DNA amplification, while methylated C residues remain unaffected (Figure 1) [38]. Analysis of bisulfite-converted DNA was previously coupled with Sanger sequencing to investigate specific DNA sequences. Nowadays, NGS allows for genome-wide methylation study, where DNA methylation sites in a single-base resolution can be detected. However, bisulfite conversion causes substantial DNA degradation [39], which may result in loss of some critical information, especially when cfDNA input is typically low. Therefore, the library preparation and treatment process should be optimized for high-quality bisulfite sequencing. 

#### 2.2.1. Whole-Genome Bisulfite Sequencing (WGBS)

**Figure 2:**
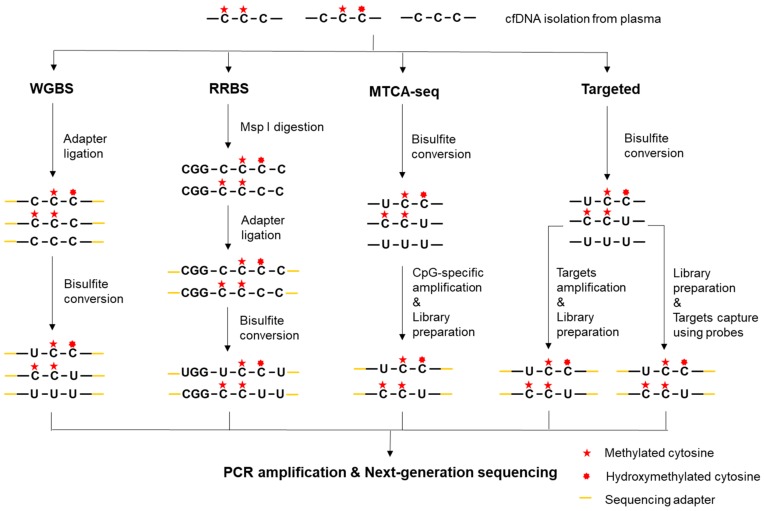
Schematic diagram of bisulfite-based cfDNA methylation profiling technologies, including whole-genome bisulfite sequencing (WGBS), reduced-representation bisulfite sequencing (RRBS), methylated CpG tandems amplification and sequencing (MCTA-seq), and targeted bisulfite sequencing.

WGBS presents the most comprehensive and informative DNA methylation profiling technology [40], which was first developed to map the human DNA methylomes [41]. The major advantage of WGBS is that the methylation state of every cytosine, including low CpG density regions and non-CpG sites (CpA, CpT, and CpC), can be detected. However, since the whole genome is targeted, the cost of WGBS is extremely high when producing high depth data. To apply WGBS for liquid biopsies, end repair and methylated adapter ligation at both ends of cfDNA fragments are performed before bisulfite treatment as it ensures amplification after bisulfite treatment (Figure 2) [42]. To address increasing demand for the analysis of low input DNA, optional methods such as single-cell bisulfite sequencing (scBS-seq) [43] and single-cell whole-genome bisulfite sequencing (scWGBS) [44] have also been developed. scBS-seq adopts a post-bisulfite adapter tagging protocol to reduce bisulfite-induced DNA loss and eliminate the need for global amplification [45], while scWGBS uses post-bisulfite, single-strand library preparation. WGBS has been applied for mapping cancer-associated cfDNA methylation in metastatic breast cancer (BC) [46]. However, as the cost of large-scale WGBS is prohibitive, a sample pooling approach was adopted. In the absence of individual sample analysis, a few samples may overshadow the other samples. As a result, the complexity of the pool is reduced. Fortunately, the costs of sequencing have continuously decreased in recent years, making WGBS more and more economically feasible.

#### 2.2.2. Reduced-Representation Bisulfite Sequencing (RRBS)

To investigate DNA methylome more cost-effectively, RRBS was developed by integrating *Msp*I digestion, bisulfite conversion, and NGS for the analysis of CpG-rich regions [47,48]. This method can detect more than 83% of CGIs in mammalian genome when the *Msp*I-digested fragments are size-selected between 40 and 220 bp [49]. Similar to MRE-seq, RRBS has relatively low coverage toward intergenic and distal regulatory elements because of the limited CpG-containing recognition sites. To apply this method for limited cfDNA, single-cell RRBS (scRRBS) has been developed and the input is significantly decreased. To avoid DNA loss, scRRBS integrates all the key RRBS reactions into a single-tube reaction so that DNA purification does not occur until the bisulfite conversion is completed (Figure 2). This is achieved by modifying the buffer system and the reaction volumes to preserve the activities of different enzymes [50,51]. Also, it is recommended that a gel-based approach is used in size selection step to thoroughly remove the primer dimers, which are more likely to occur when the input is low. Capitalizing on this strategy, scRRBS has been successfully used for methylation profiling of plasma cfDNA for the first time. Consequently, methylated haplotype analysis in plasma cfDNA has demonstrated the quantitative estimation of tumor load and tissue-of-origin mapping [52]. 

#### 2.2.3. Methylated CpG Tandems Amplification and Sequencing (MCTA-seq)

MCTA-seq is a highly sensitive technique for detecting hypermethylated CGIs [53]. In this approach, a primer that consists of a semi-random sequence, a unique molecular identifier (UMI) sequence, and an anchor sequence is used to amplify and extend the bisulfite converted DNA at the 3′-end. Compared to unmethylated CGIs and non-CGI fragments, methylated CGIs are expected to be amplified to a higher degree because of the higher methylated CpG density. Then, the methylated CpG tandem sites are selectively amplified using another primer containing the CpG tandem sequence CGCGCGG. Only the methylated CGI sequences can be further amplified and sequenced (Figure 2). This approach preferentially enriches CGIs and allows as little as 7.5 pg cfDNA input through multiple rounds of amplification. However, as MCTA-seq can only detect CpG tandem regions, it will miss the non-CpG methylation. Application of the MCTA-seq in cfDNA has identified dozens of DNA hypermethylation markers for effective detection of hepatocellular carcinoma (HCC) and colorectal cancer (CRC) [53,54]. These biomarkers, including known and novel, demonstrate a high sensitivity and specificity for disease detection at an early stage.

#### 2.2.4. Targeted Bisulfite Sequencing

Although allowing for the discovery of novel DNA methylation alterations, the methods described above are not practical for clinical applications, where a rapid turn-around time, cost-efficient methods and high depth of sequencing coverage are required [55]. Consequently, targeted methylation sequencing is more clinically pragmatic, because it is scalable, economical, and allows for a higher depth of sequencing coverage. Depending on target enrichment manners, targeted bisulfite sequencing may be categorized into the following two groups: target amplification using PCR and target enrichment using probe hybridization (Figure 2). Specific primers can be used to amplify regions of interest after the bisulfite treatment, such as *EFC#93* primers for disseminated BC [56] or *Vimentin* and *Fibulin 1* primers for HCC [57]. Alternatively, probe sequences can be designed, synthesized, and 5’-biotinylated for target enrichment of the bisulfite converted libraries. Namely, 5’-biotinylated capture probes are used to specifically pull down DNA fragments that contain target CpG sites. This method has been used for cancer detection and classification [58]. Although targeted bisulfite sequencing has been investigated for cancer diagnostics and assessment of therapeutic outcomes, this method is constrained by the relatively complicated primer and probe design for bisulfite-converted sites.

#### 2.2.5. Methylation Array

Illumina Infinium HumanMethylation450 BeadChip (HM450K) contains predesigned probes for more than 450k methylation sites that cover 96% of the CGIs [59] and dominated as the method of choice for the cancer methylome studies before the prevalence of NGS [60]. Infinium MethylationEPIC BeadChip, a further developed version, covers more than 850k CpG methylation sites, including almost all sites on the 450K array plus additional CpG sites in the enhancer regions [61]. Currently, a huge number of HM450K datasets on Gene Expression Omnibus (GEO) [62] and The Cancer Genome Atlas (TCGA) [63] have become an outstanding public resource for the discovery of novel DNA methylation markers [19,64] and the validation of new DNA methylation assays. As for liquid biopsies, the Infinium methylation array has been applied for the epigenome-wide discovery of non-invasive methylation biomarkers for CRC using a cfDNA pooling strategy [65]. The methylation data have also been used for the deconvolution of the plasma methylome for the inference of tissue origins of cfDNA [29]. However, all array-based methods have a drawback in poor genome-wide coverage of all methylation sites, resulting in the loss of other methylation contexts.

#### 2.2.6. Methylation-specific PCR (MSP)

MSP is based on the use of two distinct methylation-specific primer sets for detecting the DNA of interest. The methylated primer will amplify bisulfite converted methylated DNA and untreated DNA, while the unmethylated primer is specific for bisulfite converted DNA in an unmethylated condition [66]. Taking advantage of real-time PCR, several quantitative MSP (qMSP) protocols have been developed [67,68,69]. Moreover, methylation-sensitive high-resolution melting analysis (MS-HRM) has been developed for methylation detection [70]. These technologies have been widely used in the identification and validation of ctDNA-specific aberrant DNA methylation [71,72,73,74,75]. For example, the plasma cfDNA methylation of *SEPT9* has been identified as a biomarker for the noninvasive diagnosis of CRC and hepatocellular carcinoma [76,77]. With a significant advance on ddPCR, a droplet digital methylation-specific PCR (ddMSP) panel has been established for the cfDNA-based early detection of BC [78]. Also, methylation of *NPY* and a five-genes panel have been reported as biomarkers for metastatic CRC using ddMSP [79,80]. Yet, these individual markers only provided a limited picture of the whole tumor methylome. Therefore, a combination of multiple markers is highly recommended in a clinical setting to ensure a high sensitivity and specificity.

### 2.3. Enrichment-based methods

The strategy for enrichment-based methods is to use anti-methylcytosine antibodies or methyl-CpG binding proteins to pull-down the methylated genomic regions for subsequent analysis, while the unmethylated fractions are excluded by stringent washing. Compared to WGBS methods, these enrichment-based methods have not only shown a similar sensitivity and slightly better specificity [81] but also many other advantages. They are cost-effective because only the enriched fragments are sequenced so many more indexed samples can be pooled simultaneously for NGS. Furthermore, the enrichment approach does not involve cytosine conversion and can discriminate 5mC from 5-hydroxymethylcytosine (5hmC) due to the protein-binding specificity. However, these methods have a relatively low resolution of ≈100–300 bp, and therefore may not discriminate the exact methylation context. Additionally, these methods tend to exhibit biases toward hypermethylated regions. As the standard protocol of these methods require relatively large amount of DNA input, further optimization for the library preparation and methylation enrichment is needed for cfDNA-based studies. It is necessary to be aware that missing signal can mean an unmethylated sequence or an uncaptured sequence due to low input.

#### 2.3.1. Methylated DNA Immunoprecipitation Sequencing (MeDIP-seq)

**Figure 3:**
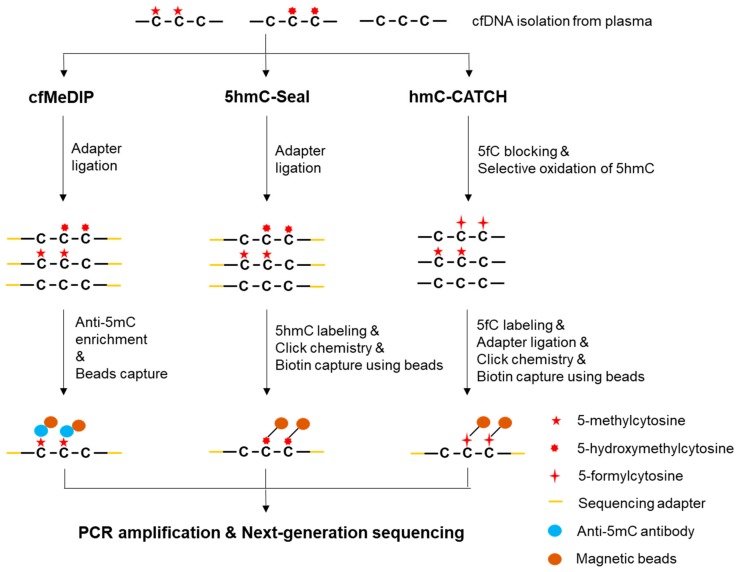
Schematic diagram of enrichment-based cfDNA methylation profiling technologies, including cell-free methylated DNA immunoprecipitation and high-throughput sequencing (cfMeDIP-seq), 5hmC-Seal, and hmC-CATCH.

MeDIP was originally developed as an approach for the immunoprecipitation of methylated DNA followed by a microarray analysis [82]. A low DNA input protocol has been reported to reduce the required input from 5000 ng to 50 ng DNA. However, using less than 50 ng DNA as an input was not recommended due to insufficient methylation enrichment [83]. To apply MeDIP-seq for low-input cfDNA in liquid biopsies, cell-free methylated DNA immunoprecipitation and high-throughput sequencing (cfMeDIP-seq) has been developed, where exogenous lambda DNA is used as a filler to increase the initial DNA input (Figure 3) [84]. The filler DNA ensures a constant antibody/DNA ratio and helps maintain a similar immunoprecipitation efficiency across different samples with different cfDNA yields, while minimizing non-specific binding and DNA loss [85]. With the help of filler DNA, the starting cfDNA can be reduced to ≈1-10 ng. Because the lambda DNA does not have sequencing adapters, and hence no subsequent amplification, the use of filler DNA would not interfere with the analysis of sequencing data. An application of the cfMeDIP-seq in a group of lung cancer patients and controls has identified a set of significant differentially methylated genes [86]. However, the sample size (n = 3) was too small and a large dataset is needed to validate the finding.

#### 2.3.2. Methyl-CpG Binding Domain Protein Capture Sequencing (MBD-seq)

Instead of immunoprecipitation, the methyl-CpG binding domain (MBD) of methyl-CpG binding proteins (MBD2 or MECP2) can be used to pull down methylated DNA fragments with the help of magnetic beads [87]. It has been shown that MBD-based enrichment outperforms MeDIP in regions with a higher CpG density and identifies the greatest proportion of CGIs [88]. Therefore, integrating MBD-seq with liquid biopsies may facilitate the discovery of ctDNA hypermethylation signatures. A study has described a low DNA input MBD-seq protocol by adjusting the DNA to beads ratio and using more incubation time and more stringent wash conditions [89]. Using this protocol, MBD-seq with a 15 ng DNA input detected 93% of the methylated loci that were reliably detected using WGBS (sensitivity) at similar levels of the false positive rate (specificity). Even with as little as 5 ng DNA, MBD-seq had a 90% of sensitivity and equal levels of specificity relative to WGBS [89]. Therefore, this low-input technology is suitable for liquid biopsy studies. Also, it is expected that the use of exogenous DNA as a filler to increase the initial input might increase the capture efficiency for MBD proteins as it does for immunoprecipitation.

### 2.4. 5-hydroxymethylation profiling

Ten-eleven translocation (TET) enzymes can oxidize 5mC to 5hmC, 5-formylcytosine (5fC), and 5-carboxylcytosine (5caC), which is known as DNA demethylation (Figure 1) [90,91]. Emerging evidence indicates that 5hmC not only acts as a relatively stable epigenetic marker in mammals [92] but also correlate with tumorigenesis and tumor progression [93]. Previously, studies have shown the reduced global 5hmC levels but increased regional 5hmC levels in various cancer tissues [94]. These observations suggest that 5hmC signatures may also be promising biomarkers for cancer diagnostics and prognostics. Due to the low frequency of 5hmC, however, the detection of 5hmC is technically more challenging. Also, bisulfite sequencing does not distinguish between 5mC and 5hmC because both are resistant to bisulfite treatment [95]. Hence, bisulfite-free 5hmC profiling methods have been developed. 

#### 2.4.1. 5hmC-Seal (aka hMe-Seal)

In 5hmC-Seal, an azide-modified glucose is first introduced by β-glucosyltransferase (β-GT) and subsequently biotinylated via click chemistry in selective chemical labeling (Figure 3) [96]. The biotinylated 5hmC is then enriched using streptavidin beads followed by NGS to determine the genomic distribution of 5hmC, where spike-in probes are adopted to test the 5hmC capture efficiency during the 5hmC-Seal assay. As 5hmC-Seal can work with ultra-low levels of starting DNA (≈5 ng) [94], this technology has been applied for liquid biopsies, where cfDNA was first ligated with sequencing adapters. The proof-of-principle global analysis of hydroxymethylome in cfDNA has been reported [97]. Since then, the 5hmC-Seal has been used to identify a genome-wide pattern of cancer-associated 5hmC changes and tissue origins of such changes in plasma cfDNA from a patient-derived xenograft mouse model [98]. The method has also been used to detect large-scale aberrant 5hmC alternations in both gene bodies and promoter regions for non-small-cell Lung Cancer (NSCLC) [99], CRC [100], HCC [101], and esophageal cancer [102]. Because the total read count is used in predefined genomic regions to estimate 5hmC level, the resolution of 5hmC-Seal is relatively low.

#### 2.4.2. hmC-CATCH

To solve the problem of low resolution, hmC-CATCH, a bisulfite-free method for the genome-wide detection of 5hmC, has been reported recently, where the single-base resolution hydroxymethylome in human has been revealed for the first time [103]. This method is based on the principle of blocking 5fC, selective oxidation of 5hmC to 5fC, chemical labeling of the newly generated 5fC, and a subsequent C-to-T transition during PCR amplification (Figure 3). Because the endogenous 5fC is first blocked and only the newly generated 5fC are detected, this technology is specific for 5hmC. To test hmC-CATCH for liquid biopsies, 5hmC enrichment profiles in cfDNA using both hmC-CATCH and 5hmC-Seal have been compared and the results showed good agreement [103]. Therefore, this technology has great potential for high resolution 5hmC signature studies.

#### 2.4.3. Hydroxymethylated DNA Immunoprecipitation Sequencing (hMeDIP-seq)

hMeDIP is modified from MeDIP and allows for specific enrichment of DNA fragments containing 5hmC [104]. This method has become an invaluable tool for determining genome-wide profiles of 5hmC. hMeDIP involves immunoselection and immunoprecipitation using anti-5hmC antibodies followed by downstream analysis such as PCR, microarray, or NGS. So far, application of hMeDIP-seq for liquid biopsies has not been reported, possibly due to the input limitation. Like cfMeDIP-seq, it is expected that application of exogenous DNA as a filler to increase the initial input may facilitate cfDNA 5hmC profiling analysis.

#### 2.4.4. Oxidative Bisulfite Conversion

Taking advantage of the fact that cytosines in 5fC and 5caC are not protected from deamination by sodium bisulfite (Figure 1), oxidative bisulfite sequencing (OxBS-seq) [105] and TET-assisted bisulfite sequencing (TAB-seq) [106] have been developed, respectively. However, as longer bisulfite treatment and oxidative environment are needed for the efficient conversion of 5mC to 5fC or 5caC, more DNA degradation and DNA damage may occur [107]. Application of these methods in liquid biopsies need further investigation.

## 3. Bioinformatics Analysis of Sequencing-Based DNA Methylation Data

**Figure 4:**
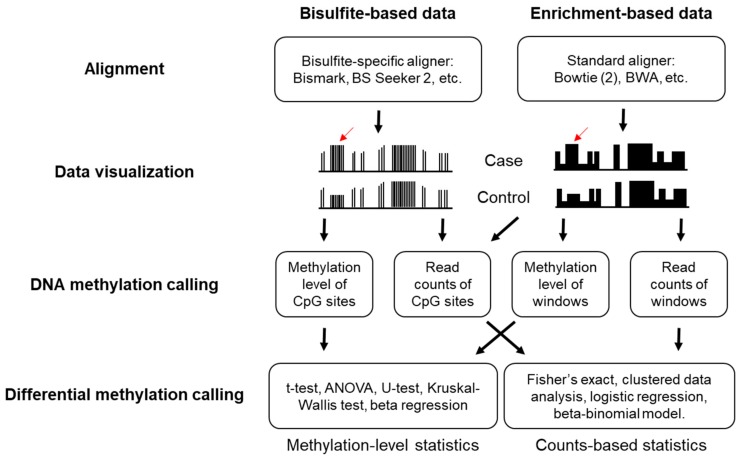
Computational pipeline for DNA methylation sequencing data analysis.

The general workflow for the bioinformatics analysis of DNA methylation sequencing data includes quality assessment of reads (FastQC [108]), adapter trimming (Trimmomatic [109], Trim Galore [110]), read alignments to a reference genome, post-alignment quality control, data visualization (UCSC Genome Browser [111], Integrative Genomics Viewer (IGV) [112], Methylation plotter [113], and Web Service for Bisulfite Sequencing Data Analysis (WBSA) [114]), quantification of the DNA methylation level or genomic coverage, and identification of differentially methylated cytosines (DMCs) or differentially methylated regions (DMRs) (Figure 4) [115,116]. Here, we will provide the analysis strategies for DNA methylation sequencing data. All these strategies are highly compatible when cfDNA is used as a starting material.

### 3.1. Alignment and Quality Controls

Standard aligners are not available for bisulfite sequencing data because the bisulfite converted DNA does not align to the reference genome. To address this issue, two algorithms have been developed for the alignment of bisulfite sequencing data: wild card algorithm (GSNAP [117]) and three-letter algorithm (Bismark [118], BS Seeker 2 [119,120]). The wild card algorithm allows both Cs and Ts in reads to map into Cs in the reference genome, while the three-letter algorithm converts all Cs in the reference genome and the reads into Ts, and thus standard aligners can be adopted [121]. Post-alignment quality control is important for bisulfite sequencing data to reliably quantify read counts and the methylation level per base. For example, base-calling quality should be checked because miscalled bases can be counted as C-T conversions. Since the end repair step in library preparation may introduce either methylated or unmethylated Cs [115], low quality bases on sequence ends should be trimmed to minimize false C-T conversions. It is critical to check the unique alignment rates and insert lengths after trimming because bisulfite treatment causes substantial DNA degradation. Incomplete bisulfite conversion also causes false positive results as unconverted unmethylated Cs are considered as methylated. To address this issue, spike-in sequences with unmethylated Cs are usually added to measure the bisulfite conversion rate. As the majority of CpGs with high inter-population differences contain common single nucleotide polymorphisms (SNPs) [122], filtering out known C/T SNPs is highly recommended. Additionally, removal of duplicate reads that align to the same genomic position arising from PCR bias is considerable. However, this is problematic in RRBS because by design reads start at the same position even if they are not PCR duplicates. Instead, one can remove regions with an unusually high coverage.

Differing from bisulfite-based methods, standard aligner (bowtie2 [123], BWA [124]) can be used for the alignment of enrichment-based sequencing data as no mutation is introduced during library preparation. In the cases of enrichment-based sequencing data, none of the post-alignment quality control issues mentioned above except duplicate reads, need to be considered. Duplicate reads are increasingly likely to occur because reads are expected to align to a smaller methylation-enriched genome, while some duplicate reads occur by chance owing to the methylation enrichment, not the PCR over-amplification. Poisson statistic has been used to determine the maximal number of duplicate reads allowed per genomic position [125].

### 3.2. DNA Methylation Calling

After a series of quality control, the methylation level of each CpG site can be calculated in the bisulfite sequencing data, which is a number ranging from 0 to 1. This is simply done by counting the number of C-T conversions and dividing the number of Cs by the sum of Cs and Ts for each C. As relative ratio, the methylation level would normalize the coverage difference at each individual CpG site, which vary dramatically due to genomic feature and amplification differences. Also, the methylation level can be easily fit into many commonly used statistics models as it is a continuous variable. However, the methylation level calculated from the CpG site with a low sequencing depth is less reliable [126]. Alternatively, analysis on the read counts or CpG coverage data may be considered, which means directly analyzing the number of methylated and unmethylated Cs at each CpG site. 

Unlike bisulfite sequencing data, data from enrichment-based methods are usually analyzed by comparing the relative abundance of fragments. Generally, the genome is divided into non-overlapped adjacent windows of a specified width and the number of read counts in each window across all samples can be used for further analysis. For analysis involving multiple samples, data normalization is crucial to remove biases between samples or different batches. Reads per kilobase million (rpkm) is a popular choice as it rescales read counts to correct for differences in both the library size and fragment length [127]. The trimmed mean of M-values (TMM) [128] and DESeq [129] calculate a scaling factor via different algorithms. Then read counts are normalized by using these scaling factors. The genomic binning function and both rpkm and TMM normalization are implemented in the R package MEDIPS [125], while DESeq normalization is implemented in the R package DESeq2. More recently, a Bayesian statistical model that transforms the methylation enrichment read counts to absolute methylation levels has been developed and is implemented in the R package QSEA [130,131]. Additionally, a non-parametric method that uses isolated CpGs to estimate sample-specific fragment size distributions for the estimation of the CpG coverage of each CpG site has also been developed and is implemented in the R package RAMWAS [132,133]. 

### 3.3. Determination of Differential Methylation

Following methylation calling, statistical tests can be employed to identify differential methylation between cases and controls. Differential methylation in cancer means CpG sites or regions that have different DNA methylation patterns between cancer patients and healthy individuals. For a comparative analysis of the methylation level on a CpG site or region between multiple samples, standard statistical methods such as t-test, ANOVA, nonparametric test (Mann–Whitney U test and Kruskal-Wallis test), and beta regression may be used. Considering the distribution of methylation level among the study population is unknown, a nonparametric test is more preferentially adopted in methylation studies (R package BSmooth [134] and limma [135]). ANOVA is based on linear models and allows for a multiple-group comparison (R package Minfi [136]). Thus, it is more suitable in a clinical setting as it allows for the incorporation of covariates. For read counts analysis, Fisher’s exact (or chi-square) test, clustered data analysis [137], logistic regression [138], and the beta-binomial model [139,140,141] may be used. Fisher’s exact test is the method of choice when replicates are not available, however, it does not take the biological variability of methylation into consideration due to the pooling analysis. Regression methods also allow adding covariates, such as age and sex into the tests, which are shown to be influential on the methylation level [142]. Among these models, the beta-binomial model is the best method for balanced sensitivity and specificity in DMC detection [126].

As the methylation level between neighboring CpG sites are potentially positively correlated, a combination of multiple adjacent CpG sites into a defined region called DMR can reduce the number of hypothesis tests and thereby improve the statistical power [115]. DMRs can be determined by clustering nearby CpGs or DMCs, or by applying segmentation methods to segment the differential CpGs into hyper/hypo-methylated regions [143]. DMRs can also be defined based on predefined regions, such as gene promoters and CpG islands, or adjacent CpG sites within user-defined non-overlapped windows across the whole genome [144]. To better measure weak methylation differences, increasing the biological replicates and sequencing depth present a good strategy to obtain more robust *p*-values. The inherent limitation of high dimensional data is false positive. Therefore, statistical results must be subjected to multiple testing corrections. Among all options, Bonferroni and false discovery rate (FDR) are the most commonly used. Comprehensive evaluation of almost all tools and statistical methods for identifying DMRs for DNA methylation sequencing data has been summarized [126,145,146,147,148,149]. After the calling of DMRs, the regions of interest often need to be integrated with genome annotation datasets, which allows for determining whether the DMRs are related to genes and gene regulatory regions. The R package Genomation [150] and ChIPpeakAnno [151] are good annotation tools to be recommended.

### 3.4. Identification of Tumor-Specific Methylation Profile

Due to the high background of normal cells-derived cfDNA, the ctDNA concentration in cfDNA is generally low in cancer patients. Therefore, it is challenging to identify ctDNA methylation, especially in early-stage cancer. One common strategy is to use the methylation profile of tumor-free peripheral blood mononuclear cells (PBMCs) as a negative control. By comparing DMRs between cancer cfDNA and healthy cfDNA to those between cancer cfDNA and PBMC genomic DNA, the shared regions are considered to be tumor-specific DMRs. This strategy has been applied in many studies that identified the ctDNA methylation signatures [19,84,97,98]. Additionally, a reference-based deconvolution algorithm has been developed for correcting cell-type heterogeneity for studying methylation [152]. This algorithm allows for the recovery of the original signal from a mixture of signal sources by using reference datasets. Therefore, it is suitable for the deconvolution of data from heterogeneous samples, including cfDNA, when tissue-specific and cancer-specific methylation data are available. The deconvolution algorithm has been successfully applied to estimate ctDNA content and differentiate tissue-of-origin in cfDNA of patients with lung or colorectal cancer [52]. Recently, probabilistic models have been formulated to identify ctDNA methylation. CancerLocator, a tool for non-invasive cancer diagnosis and tissue-of-origin prediction, is based on such a model [153]. By using Infinium HM450K data from TCGA, CancerLocator identified many CpG cluster features that have significant methylation variation across cancer and normal samples, as well as modeled methylation levels in different cancer types. Thus, the ctDNA burden and the likelihood of the presence of a specific cancer type can be inferred based on the methylation data of informative CpG clusters. A further developed version, CancerDetector, adopts the joint methylation states of multiple adjacent CpG sites on an individual sequencing read and jointly deconvolutes the tumor fraction across all markers, has achieved a high sensitivity and specificity in detecting ctDNA methylation [154]. The Bayesian hierarchical model and methylation haplotype analysis share a similar strategy that enables information sharing across a cluster of neighboring CpG sites in order to enhance the statistical power [52,130,139].

## 4. Current Challenges and Future Directions

In the early years of development, whole blood (buffy coat) DNA was preferentially used as a starting material. However, the high background of the hematopoietic cell genome may cause a false positive detection of cfDNA-specific DMRs. Later, plasma was proven to be a superior source of cfDNA owing to the lower background levels of wild-type DNA [155]. Although superior, the combination of limiting cfDNA fragments and low ctDNA fraction in a typical early-stage cancer plasma sample still restrict the detection of methylation signatures. One should be aware that if a certain region of DNA is not present in the sample, no target enrichment technique can retrieve it. Given the extremely low ctDNA content, each sample is expected to contain a low copy number of tumor genome equivalents. Therefore, the regions with a low sequencing coverage should be considered as a potential tumor signal, rather than filtering out as low quality reads. Unfortunately, most analytical pipelines do not take such a scenario into consideration and remove low-coverage regions as part of routine quality control. This may sound reasonable for recurrence detection in an advanced stage cancer, in which tumor burden is high. However, it is obviously improper for early stage cancer since the tumor burden is extremely low.

The use of NGS in epigenetic studies has significantly facilitated our discovery of DNA methylation biomarkers. However, these studies also face some challenges, including the lack of a uniform pipeline for both experimental and computational methods. Thus, different laboratories may generate different set of biomarkers from the same type of disease or the same set of samples, and in some cases, different interpretations for the same datasets. To eliminate the inconsistency arising from tumor heterogeneity and distinctive analytical methodologies, integration of different assays and multiple biomarkers may be considered as a strategy of choice. It is believed that the combination of various methylation assays will ensure the generation of more reliable biomarkers or novel assays. For example, the high sensitivity of cfMeDIP-seq for low DNA input has been validated by comparing the results with traditional MeDIP-seq, RRBS, and WGBS [84]. Additionally, the combination of multiple methylation markers will help to achieve a higher analytical performance. For instance, the recent assays for the early detection of BC and monitoring of treatment response in CRC used multiple methylation signatures to improve outcome [78,80]. Furthermore, the integration of cfDNA methylation analysis with other aberrant cfDNA alternations assays, such as copy number variations and point mutations, may also improve the diagnostic sensitivity and specificity. 

Although promising, the integrated strategy will produce more complicated data, which requires a more sophisticated analytical algorithm. With the rapid development of new computational technologies, the use of machine learning for diagnostic and therapeutic decision- making is receiving more popularity. For example, artificial intelligence systems have been adopted for methylation analysis in recent studies [84,156,157]. It is expected that machine learning will allow for the identification of trends and cancer-specific patterns with ease. However, machine learning has many obstacles. First, it requires massive training data sets. For a cancer diagnosis, more cohort information, such as sex, age, cancer type and stage, as well as diverse environmental factors (sample collection and storage), are necessary. Second, labeling and annotating the training data is a time-consuming process. Furthermore, computer program skills are also required for scientists to comprehensively analyze and interpret the vast amount of data. Therefore, significant effort is still needed to fully realize clinical application of cfDNA methylation markers in the cancer detection and outcome prediction.

Besides cfDNA methylation, other epigenetic biomarkers have also been explored for liquid biopsies. With the rapid development of cell sorting technologies, investigation of the methylome in circulating tumor cells (CTCs) has become possible [158]. A fundamental connection between phenotypic features of CTCs and DNA methylation dynamics in stemness and metastasis has been identified recently [159]. More knowledge regarding the CTC methylome remains to be further explored. Meanwhile, cell-free RNA methylation and cfDNA fragmentation patterns also deserve more research attentions in the future [160,161].

## 5. Conclusions

This review discussed the feasibility of current DNA methylation profiling methods in liquid biopsy applications and how technical issues are addressed by improvements in technology. We summarized the advantages and disadvantages of each technique in Table 1 as guidance toward selecting the experimental method that best fits the research topic. Whole-genome methylation profiling methods are more suitable for lab research, such as discovery of novel biomarkers, while targeted and locus-specific methods are more applicable for clinical settings. Currently, most cfDNA methylation studies are based on bisulfite conversion, while enrichment-based methods and the 5hmC profiling of cfDNA are beginning to show their potential. However, these approaches may result in the loss of key methylation information since bisulfite treatment causes substantial DNA degradation and methylation enrichment recovers only a small fraction of the total DNA input. Hence, integrating different methylation profiling assays will provide complementary information and minimize the risk of missing significant signatures, ensuring generation of more reliable biomarkers. Also, joint analysis of multiple cancer methylation signatures is believed as a promising strategy for improving the diagnostic outcome. This review also provided a brief overview of methylation sequencing data analysis. Currently, most liquid biopsy studies adopted the same methods as tissue-based data analysis, while the deconvolution of data from heterogeneous cfDNA is gaining more attention. The invention of novel analysis methodologies that allow for the discrimination of ctDNA methylation signal is clearly needed. Future applications will be facilitated using artificial intelligence and machine-learning algorithms to identify clinically meaningful patterns in the data. Particular attention should be paid to ameliorate the cfDNA extraction procedures for high quality cfDNA, optimizing library preparation and methylation treatment methods for low input cfDNA, increasing the sequencing depth for accurate methylation calculations, and exploiting analytical methodologies for ctDNA-specific methylation detection.

## Figures and Tables

**Table 1:** Strengths and weaknesses of all major methylation assays for liquid biopsies

Class	Technology	Strength	Weakness	Cost
**Restriction enzyme-based**		**-High CGI coverage**	**-Low resolution** **-Limited to regions in proximity to restriction enzyme sites**	
	qPCR or ddPCR	-Allows ultra-low DNA input -Easy primer design	-Loci-specific studies only	Low
**Bisulfite-based**		**-Single-based resolution**	**-Substantial DNA degradation during bisulfite treatment** **-Cannot discriminate between 5mC and 5hmC**	
	WGBS	-The most comprehensive profiling of the whole methylome	-Relatively low sequencing depth	High
	RRBS	-High CGIs coverage	-Limited to regions in proximity to restriction enzyme sites	Moderate
	MTCA-seq	-High CGIs coverage	-Limited to CGIs and might decrease other methylation backgrounds	Moderate
	Targeted	-Detect target CpG sites at high coverage	-Complicated primer or probe design	Low
	Microarray	-Pre-designed panel covering hotspot methylation	-Low genome-wide coverage of CpGs	Low
	qMSP or ddMSP	-Allows ultra-low DNA input	-Loci-specific studies only -Complicated primer or probe design	Low
**Enrichment-based**		**-No mutation introduced**	**-Low resolution** **-Biased toward hypermethylated regions**	
	MeDIP-seq	-Antibody is specific to 5mC	-Less sensitive in regions with high CpG density than MBD-seq	Moderate
**5hmC profiling**		**-Specific to 5hmC**	**-High sequencing depth is required as 5hmC has a low abundance**	
	5hmC-Seal	-Ensures accurate capture of DNA containing 5hmC	-Low resolution	Moderate
	hmC-CATCH	-Single-based resolution	-Oxidative environment would cause DNA damage	Moderate

**Abbreviations**: CGI: CpG island. CpG: 5′-C-phosphate-G-3′. qPCR: Quantitative polymerase chain reaction. ddPCR: Droplet digital polymerase chain reaction. qMSP: Quantitative methylation-specific PCR. ddMSP: Droplet digital methylation-specific PCR. MBD: Methyl-CpG binding domain.
